# Epoxy Vitrimers: The Effect of Transesterification Reactions on the Network Structure

**DOI:** 10.3390/polym10010043

**Published:** 2018-01-03

**Authors:** Facundo Ignacio Altuna, Cristina Elena Hoppe, Roberto Juan José Williams

**Affiliations:** Institute of Materials Science and Technology (INTEMA), University of Mar del Plata and National Research Council (CONICET), Av. J. B. Justo 4302, 7600 Mar del Plata, Argentina; faltuna@fi.mdp.edu.ar (F.I.A.); hoppe@fi.mdp.edu.ar (C.E.H.)

**Keywords:** epoxy-acid reactions, epoxy vitrimers, fragment approach, network structure, statistical analysis, transesterification reactions

## Abstract

Vitrimers are covalently crosslinked polymers that behave as conventional thermosets below the glass transition temperature (T_g_) but can flow above a particular temperature, T_v_ > T_g_, by bond exchange reactions. In epoxy vitrimers, transesterification reactions are responsible for their behavior at T > T_v_ that enables flow, thermoforming, recycling, self-healing and stress relaxation. A statistical analysis based on the fragment approach was performed to analyze the evolution of the network structure of epoxy vitrimers during transesterification reactions. An analytical solution was obtained for a formulation based on a diepoxide and a dicarboxylic acid. A numerical solution was derived for the reaction of a diepoxide with a tricarboxylic acid, as an example of the way to apply the model to polyfunctional monomers. As transesterification acts as a disproportionation reaction that converts two linear fragments (monoesters) into a terminal fragment (glycol) and a branching fragment (diester), its effect on network structure is to increase the concentration of crosslinks and pendant chains while leaving a sol fraction. Changes in the network structure of the epoxy vitrimer can take place after their synthesis, during their use at high temperatures, a fact that has to be considered in their technological applications.

## 1. Introduction

Fatty acids have been employed in epoxy formulations for varnishes and coatings since the middle of the last century. The esters generated provide excellent properties to the resulting materials, including adhesion, flexibility, water-resistance and brushing and grinding ease. Unsaturated fatty acids are used for air-dried coatings while saturated fatty acids are used in sub-stoichiometric ratios and reaction of the epoxy excess is performed adding conventional hardeners such as polyamines. Modern high performance-coatings are based on the use of polycarboxylic acids as hardeners of acrylate-epoxy monomers [1].

**Scheme 1:**
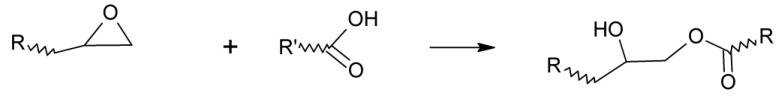
Addition reaction of a carboxylic acid to an epoxy group.

The addition reaction of an epoxy group with a carboxylic acid either uncatalyzed or employing a variety of different catalysts, such as tertiary amines or triphenyl phosphine, generates a β-hydroxyester as shown in Scheme 1.

**Scheme 2:**
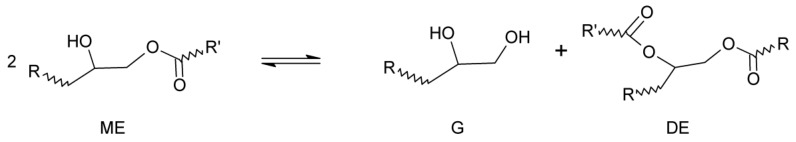
Convertion of monoesters (ME) into a glycol (G) and a diester (DE).

Secondary reactions can take place such as the epoxy homopolymerization initiated by the same catalysts or the esterification of the OH groups with the carboxylic acid leading to a new ester group and water as a reaction product. In turn, epoxy groups can be hydrolyzed with the water generated. The sequence of these reactions was clearly established by Dušek and co-workers [2]. They studied the reaction of a monoepoxide (phenyl glycidyl ether) with a monocarboxylic acid (hexanoic acid), catalyzed by triethylamine. For every stoichiometric ratio investigated, they found that the epoxy-carboxylic acid reaction took place first up to an almost complete conversion. After completion of this reaction, homopolymerization was observed in formulations with epoxy excess and condensation esterification in the presence of a carboxylic acid excess. However, they observed that the concentration of the monoester (ME) produced by the epoxy-acid reaction did not remain constant but decreased to a final constant value after keeping the system for several hours at the selected reaction temperature. The reason was the partial conversion of the monoester into a glycol (G) and a diester (DE) by a transesterification reaction until equilibrium was attained (Scheme 2).

Transesterification reactions were well known in organic chemistry but their presence in the epoxy-acid chemistry had not been previously emphasized. A conclusion of this study was that in a polyfunctional system this reaction may significantly affect the network structure by breaking the chains and giving place to new crosslinks.

The significance of the experimental findings of Dušek and co-workers remained dormant for almost three decades until the group of Leibler in Paris discovered that transesterification reactions gave unusual properties to the epoxy-carboxylic acid networks giving rise to a new class of materials called epoxy vitrimers [3,4,5]. They realized that, in the temperature range where transesterification reactions take place, the crosslinked epoxy is able to interchange fragments of the network structure. This takes place by production and recombination of glycol and diester fragments enabling the interchange of monoester fragments located in different positions of the network. The possibility of interchanging fragments of the network structure enables flow, thermoforming, recycling, self-healing and stress relaxation among several other desirable properties. This discovery rapidly led to the design of a set of new materials for advanced technological applications.

**Figure 1:**
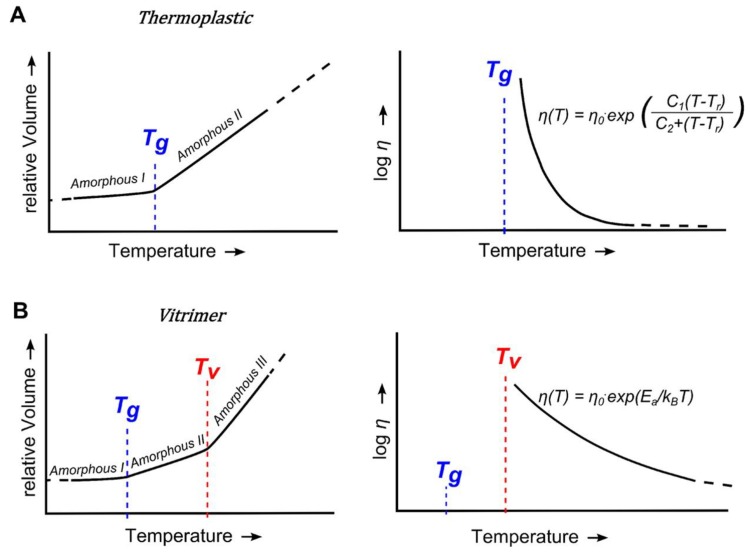
(**A**) Left: V-T characteristics of an amorphous thermoplastic polymer. Right: At T > T_g_, viscosity follows the WLF equation; (**B**) Left: V-T characteristics of a vitrimer. At T > T_v_, bond exchange reactions enable flow. Right: At T > T_v_, viscosity follows an Arrhenius equation. Reprinted with permission from Capelot et al. [5].

The behavior of a vitrimer can be visualized in Figure 1.

Transesterification reactions are in fact activated at T > T_g_ where β-hydroxyester segments acquire enough mobility. However, the observation of flow depends on the timescale of the experiment. The arbitrary temperature T_v_ is defined at a viscosity level of 10^12^ Pa.s, characteristic of a solid-to-liquid transition. At T > T_v_, the flow is governed by the kinetics of transesterification reactions that follow an Arrhenius equation. This keeps viscosity at high values in a broad temperature range. This behavior enables the thermoforming of complex shapes and is characteristic of strong glasses like inorganic silica glass. This is the origin of the identification of these materials as epoxy vitrimers.

Following the seminal papers of Leibler and coworkers [3,4,5], an explosion in the literature of epoxy vitrimers based on the epoxy-acid chemistry took place, particularly focused on bio-based formulations [6], action of transesterification catalysts [7]; characterization and theoretical modeling of viscoelastic and mechanical properties and welding behavior [8,9,10,11,12,13,14]; self-healing, reprocessing and recycling [15,16,17,18]; remote and multi-stimuli activation [19,20,21,22,23,24]; and silica-reinforced [25] and other advanced materials [26,27,28,29,30,31,32]. The behavior of epoxy vitrimers based on the epoxy-acid anhydride chemistry has also been analyzed [3,4,33,34]. However, the epoxy-anhydride reaction produces ester groups, making it necessary to generate the OH groups needed to promote transesterification by other set of reactions (homopolymerization of an epoxy excess [35], or alcohol or water addition to the initial formulation). Epoxy-acid formulations employing a high epoxy-excess are also employed to manufacture paramagnetic composites [36,37]. In what follows, we will restrict our analysis to epoxy vitrimers generated by epoxy-carboxylic acid formulations.

The focus of the recent literature in the field is placed on the relevant properties of the materials generated and their possible technological applications. Even if the occurrence of transesterification reactions is always indicated as the basis of their behavior, the effect of these reactions on the network structure, mentioned in the pioneering study of Dušek and co-workers [2], is practically not considered at all. In this article, we will theoretically analyze this effect for two different types of networks: those based on the stoichiometric reaction of a dicarboxylic acid (A_2_) with a diepoxide (B_2_) and those arising from the stoichiometric reaction of a tricarboxylic acid (A_3_) with a diepoxide (B_2_). Again, we will use as a starting point a (rather) forgotten study of the group of Dušek[38] and we will expand it to analyze the effect of transesterification reaction on the evolution of average statistical parameters of both types of epoxy networks. Some concepts related to the modification of average statistical parameters of the network during the processing of an epoxy vitrimer will be discussed. 

## 2. Equilibrium in Transesterification Reactions

Transesterification reactions of β-hydroxyesters are reversible and lead to an equilibrium state [2,38]:

(1) 2 ME=G+DE

The equilibrium constant is given by:

(2) $$K=\frac{\left(G\right)\left(\mathrm{DE}\right)}{{\left(\mathrm{ME}\right)}^{2}}\;=\frac{{\left(G\right)}^{2}}{{\left(\mathrm{ME}\right)}^{2}}$$

where (G) = (DE). It is convenient to express equilibrium in terms of the conversion in the transesterification reaction (x): (ME) = (ME)_0_·(1 − x), and (G) = (DE) = (ME)_0_·x/2. Therefore, at equilibrium:

(3) $$K^{1/2}=\frac{x_{\mathrm{eq}}}{2\left(1-x_{\mathrm{eq}}\right)}$$

or

(4) $$x_{\mathrm{eq}}=\frac{2\cdot K^{1/2}}{\left(1+2\cdot K^{1/2}\right)}$$

An experimental value of K = 0.15 ± 0.05 was reported for the model system based on the reaction product of phenyl glycidyl ether and hexanoic acid after a prolonged heating at 110–120 °C, employing tributylamine as catalyst [38]. This gives x_eq_ = 0.44, meaning that the concentrations of glycol and diester are significant when equilibrium is attained. In the analysis of the evolution of average statistical parameters of the networks, we will analyze trends up to an esterification conversion, x = 0.5, to cover the range of the equilibrium value and considering that the value of the equilibrium conversion might vary with temperature.

## 3. Statistical Analysis of Transesterification in a Stoichiometric A_2_ + B_2_ (Dicarboxylic Acid + Diepoxide) Formulation

Several formulations of epoxy vitrimers reported in the literature are based on the reaction of stoichiometric amounts of a diepoxide (usually diglycidyl ether of bisphenol A, DGEBA) with a dicarboxylic acid such as adipic (C6), suberic (C8), azelaic (C9) or sebacic (C10) acids. In most of these studies, authors verify that a gel is formed by swelling the epoxy vitrimer in an appropriate solvent. The formation of a gel is simply due to the generation of crosslinks by transesterification reactions [38].

Before performing a theoretical analysis of the network structure produced by transesterification, it is convenient to analyze experimental results obtained in a stoichiometric system of DGEBA and azelaic acid catalyzed by tributylamine [38]. The reaction was carried out at 110 °C. After about 6–6.5 h, the conversion in the epoxy-carboxylic acid reaction was higher than 0.95 and gelation was experimentally observed. At high conversions of epoxy and carboxylic acid groups, their reaction rate slows down attaining a comparable rate than the one of transesterification. The linear oligomers produced by the epoxy-carboxylic acid reaction are branched by transesterification leading to the formation of a gel. At about 8 h reaction, the residual concentration of free epoxy groups was very low (about 1–2% of their initial value and comprised within the experimental detection error). The increase in crosslink density was determined by measuring the elastic modulus of the gel as a function of the reaction time at 110 °C. An asymptotic value was obtained after about 60–100 h, showing that transesterification reactions were very slow at the selected reaction conditions. The gel fraction attained an asymptotic value of 83% early in the transesterification reaction.

A statistical treatment of network formation was performed using the tree-like model and employing cascade substitution [38]. A numerical solution enabled the prediction of the sol fraction and the concentration of crosslinks for advanced conversions in the epoxy-carboxylic acid reaction (x_E_ = 0.95, 0.99 and 1) and variable conversions (x) in the transesterification reaction. Here, the statistical analysis is performed using the fragment approach [39], a method that has been applied to predict parameters of different systems: epoxy-amine networks with simultaneous or subsequent polyetherification [40,41], homopolymerization of epoxides initiated by tertiary amines [42], polymer networks generated by living polymerization [43], polyurethane networks [44], free-radical polymerizations [45,46], cyclotrimerization of dicyanates [47,48], networks produced by stepwise and chainwise chemistries [49], epoxy-anhydride networks [50,51], epoxy monomers crosslinked with hyperbranched poly(ethyleneimine)s [52], and epoxy-thiol networks [53]. The use of this method led to simple analytical equations for the gel conversion, the concentration of crosslinks and the sol fraction.

**Figure 2:**
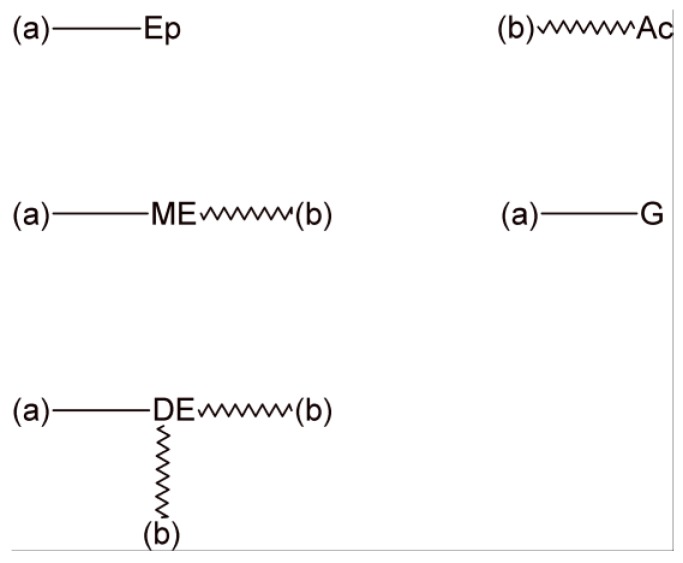
The five fragments present in the polymer structure at arbitrary conversions in the epoxy-carboxylic acid reaction (x_E_) and in the transesterification reaction (x). Bonds (a) represent half of a diepoxide molecule and bonds (b) represent half of a dicarboxylic acid molecule. The network is built-up by joining bonds (a) to bonds (a) and bonds (b) to bonds (b) with a probability determined by the concentration of corresponding fragments.

At any conversion of the epoxy-carboxylic acid reaction (x_E_) and in the transesterification reactions (x), the polymer structure is composed by the five fragments shown in Figure 2.

(Ep) represents unreacted epoxy groups with a relative concentration given by (Ep)/(Ep)_0_ = 1 − x_E_, where (Ep)_0_ is the initial molar concentration of epoxy groups. The molar mass of this fragment is M_E_/2, where M_E_ is the molar mass of the diepoxide monomer. The monoester (ME) represents fragments where the epoxy group reacted with a carboxylic acid but was not converted to transesterification products. Its relative concentration is (ME)/(Ep)_0_ = x_E_(1 − x) and its molar mass is M_E_/2 + M_A_/2, where M_A_ is the molar mass of the dicarboxylic acid monomer. The glycol (G) and diester (DE) are the fragments produced by transesterification of reacted epoxy groups. Their relative concentrations are given by (G)/(Ep)_0_ = (DE)/(Ep)_0_ = x_E_·x/2. The molar mass of the G fragment is given by M_E_/2 + 18 while the one of the diester fragment is M_E_/2 + M_A_ − 18. Fragments with unreacted carboxylic acid groups have a relative concentration (Ac)/(Ep)_0_ = (1 − x_E_) and a molar mass equal to M_A_/2.

To produce gelation, it is necessary to advance the transesterification to a specific conversion x_gel_ that depends on the previous conversion of the epoxy-carboxylic acid reaction, x_E_. The gel conversion can be calculated from the average masses attached to bonds (a) and (b):

W(a) = Σ [probability of joining a bond (a) in fragment (i)] [mass of fragment (i) + average mass attached to fragment (i) when leaving it from the remaining bonds]

W(b) = Σ [probability of joining a bond (b) in fragment (i)] [mass of fragment (i) + average mass attached to fragment (i) when leaving it from the remaining bonds]

This gives:

(5) $$W\left(a\right)=\left(1-x_{E}\right)\frac{M_{E}}{2}+x_{E}\left(1-x\right)\left[\frac{M_{E}}{2}+\frac{M_{A}}{2}+W\left(b\right)\right]+\frac{x_{E}}{2}x\left[\frac{M_{E}}{2}+18\right]+\frac{x_{E}}{2}x\left[\frac{M_{E}}{2}+M_{A}-18+2\cdot W\left(b\right)\right]$$

(6) $$W\left(b\right)=x_{E}\left(1-x\right)\left[\frac{M_{E}}{2}+\frac{M_{A}}{2}+W\left(a\right)\right]+x_{E}\cdot x\left[\frac{M_{E}}{2}+M_{A}-18+W\left(a\right)+W\left(b\right)\right]+\left(1-x_{E}\right)\frac{M_{A}}{2}$$

From Equations (5) and (6), we get:

(7) $$W\left(b\right)=\frac{f\left(M_{i},{\;x}_{E},x\right)}{\left[1-x_{E}\left(x+x_{E}\right)\right]}$$

At the gel conversion, x_gel_, W(b) → ∞ and W(a) → ∞. Therefore, the gel conversion is given by:

(8) $$x_{E}\left(x_{\mathrm{gel}}+x_{E}\right)=1$$

or

(9) $$x_{\mathrm{gel}}=\frac{1}{x_{E}}-x_{E}$$

The minimum epoxy-carboxylic acid conversion to generate a gel by transesterification is x_E_ = 0.618, making it necessary to attain complete transesterification (x = 1) to obtain a gel. However, the maximum transesterification conversion is limited by equilibrium. Assuming an equilibrium conversion, x_eq_ = 0.44, the necessary epoxy-carboxylic acid conversion to generate a gel is x_E_ = 0.804. In practice, the epoxy-carboxylic acid reaction attains very high conversions before observing any evidence of transesterification. For x_E_ = 0.95, x_gel_ = 0.103, for x_E_ = 0.98, x_gel_= 0.040 and for x_E_ = 1, a giant macromolecule is generated before the beginning of transesterification (x_gel_ = 0).

For x > x_gel_, average statistical parameters may be calculated employing the same fragments depicted in Figure 2. We will derive general analytical equations valid for any value of x_E_ comprised in the gelation region and will illustrate the results for x_E_ = 0.98 and x_E_ = 1 (limiting case where the epoxy-carboxylic acid and transesterification are strictly consecutive reactions).

Let us call F(a) and F(b) the probability of having finite continuations when looking out of bonds (a) and (b), respectively.

F(a) = Σ[probability of joining fragment i by a bond (a)](probability to have a finite continuation from fragment i)

F(b) = Σ[probability of joining fragment i by a bond (b)](probability to have a finite continuation from fragment i)

This leads to:

(10) $$F\left(a\right)=\left(1-x_{E}\right)+x_{E}\left(1-x\right)F\left(b\right)+\frac{x}{2}x_{E}+\frac{x}{2}x_{E}\cdot F{\left(b\right)}^{2}$$

(11) $$F\left(b\right)=x_{E}\left(1-x\right)F\left(a\right)+x_{E}\cdot x\cdot F\left(a\right)\cdot F\left(b\right)+\left(1-x_{E}\right)$$

From Equations (10) and (11), we get

(12) $$A\cdot F{\left(b\right)}^{3}+B\cdot F{\left(b\right)}^{2}+C\cdot F\left(b\right)+D=0$$

where

(13) $$A=\left(\frac{1}{2}\right)x^{2}\cdot x_{E}^{2}$$

(14) $$B=\left(\frac{3}{2}\right)x\left(1-x\right)x_{E}^{2}$$

(15) $$C=x_{E}^{2}{\left(1-x\right)}^{2}+x\cdot x_{E}\left[1-x_{E}+\left(\frac{1}{2}\right)x\cdot x_{E}\right]-1$$

(16) $$D=1-x_{E}+x_{E}\left(1-x\right)\left[1-x_{E}+\left(\frac{1}{2}\right)x\cdot x_{E}\right]$$

Notice that F(b) = 1 is a root of Equation (12) that has only physical sense in the pregel stage. Eliminating this root from the cubic equation leads to a quadratic equation that can be analytically solved giving a positive root comprised between 0 and 1 and a negative root without physical sense. The positive root is given by:

(17) $$F\left(b\right)=\frac{1}{2\cdot x}\left\{{\left[{\left(3-2\cdot x\right)}^{2}+4\cdot x^{2}\left(\frac{D}{A}\right)\right]}^{\frac{1}{2}}-\left(3-2\cdot x\right)\right\}$$

and F(a) can be calculated from Equation (10).

**Figure 3:**
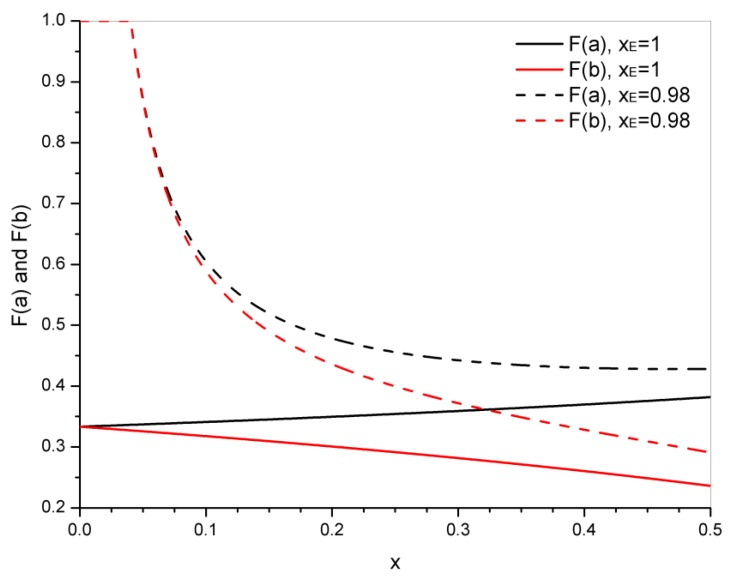
Variation of probabilities F(a) and F(b) in the 0–0.5 conversion range of the transesterification reaction, for x_E_ = 0.98 and x_E_ = 1.

It is interesting to visualize the variation of both probabilities in the 0–0.5 conversion range for x_E_ = 0.98 and x_E_ = 1 (Figure 3). In the case of x_E_ = 0.98, F(a) = F(b) = 1 up to x = x_gel_ = 0.0404 and then they both decrease with the extent of transesterification. For x_E_ = 1, both probabilities start from the same value (1/3) and F(a) increases slightly with conversion due to the predominant effect of the increase in the concentration of glycol, while F(b) decreases with conversion due to the increase in the connection of branching points to the gel.

Branching points of the network are directly given by diester units which evolve linearly with conversion, (DE) = x_E_(x/2)(Ep)_0_. Crosslinks (X_3_) are the fraction of these units with an infinite continuation to the gel from the bond (a) and both bonds (b):

(18) $$\left(X_{3}\right){=x}_{E}\left(\frac{x}{2}\right){\left(\mathrm{Ep}\right)}_{0}\left[1-F\left(a\right)\right]{\left[1-F\left(b\right)\right]}^{2}$$

**Figure 4:**
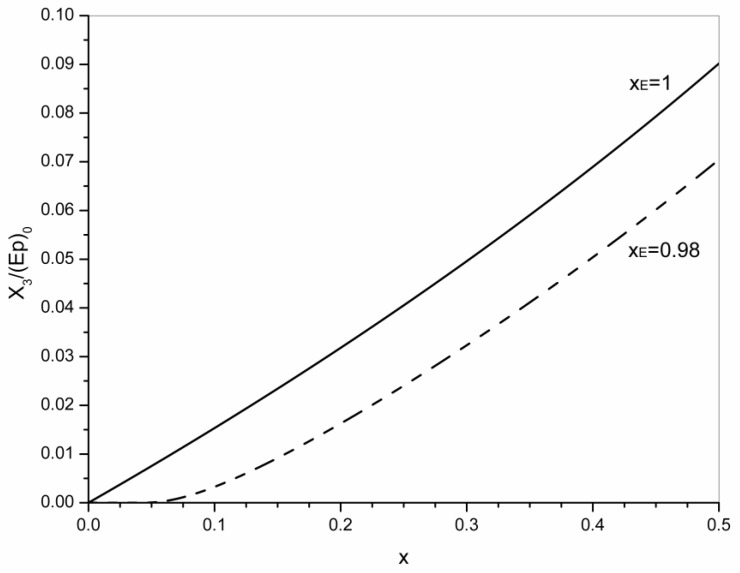
Evolution of the concentration of crosslinks during transesterification in the 0–0.5 conversion range, for x_E_ = 0.98 and x_E_ = 1.

Figure 4 shows the evolution of the concentration of crosslinks, (X_3_)/(Ep)_0_, in the 0–0.5 conversion range for x_E_ = 0.98 and x_E_ = 1. The concentration of elastic chains is (3/2)(X_3_).

For x_E_ = 1, the concentration of crosslinks increases from the beginning of transesterification. Due to the opposite variations of F(a) and F(b), only a slight departure of linearity is observed. For x_E_ = 0.98, the concentration of crosslinks begins to increase from x_gel_ and it exhibits a larger departure from linearity. This evidences the significant effect of the conversion attained in the epoxy-carboxylic acid reaction on the statistical parameters in the postgel stage.

The sol fraction can be calculated as:

(19) $$W_{\mathrm{sol}}=W_{\mathrm{Ep}}\cdot F\left(a\right)+W_{\mathrm{ME}}\cdot F\left(a\right)\cdot F\left(b\right)+W_{G}\cdot F\left(a\right)+W_{\mathrm{DE}}\cdot F\left(a\right)\cdot F{\left(b\right)}^{2}+W_{\mathrm{Ac}}\cdot F\left(b\right)$$

where the mass fractions are given by W_Ep_ = (1 − x_E_)(M_E_/2)/M_T_; W_ME_ = x_E_(1 − x)[(M_E_/2) + (M_A_/2)]/M_T_; W_G_ = (x/2)x_E_[(M_E_/2) + 18]/M_T_; W_DE_ = (x/2)x_E_[(M_E_/2) + M_A_ − 18]/M_T_; and W_Ac_ = (1 − x_E_)(M_A_/2)/M_T_. The total mass is M_T_ = (M_E_/2) + (M_A_/2) and the summation of mass fractions equals 1.

**Figure 5:**
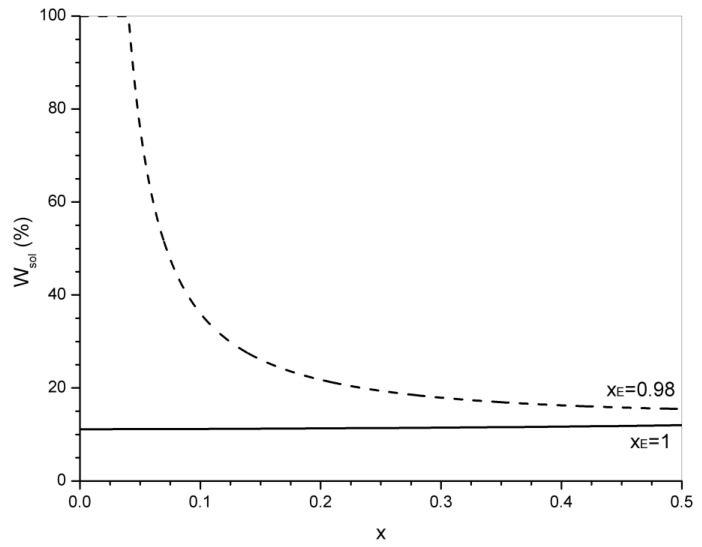
Evolution of the sol fraction in the 0–0.5 conversion range of transesterification, for x_E_ = 0.98 and x_E_ = 1.

It is interesting to compare predicted values with those experimentally found for a stoichiometric formulation of DGEBA and azelaic acid [38], M_E_ = 340 g/mol and M_A_ = 188 g/mol. Figure 5 shows the evolution of the predicted sol fractions in the 0–0.5 conversion range for x_E_ = 0.98 and x_E_ = 1. For x_E_ = 1, the sol fraction remains practically constant from the beginning of the transesterification reaction (it varies from 11.1% to 12.0%) in the whole conversion range. For x_E_ = 0.98, the sol fraction decreases rapidly at the gel conversion and more slowly at higher conversions. At conversions close to equilibrium (assumed as x_eq_ = 0.44), the predicted sol fraction is equal to 15.9% which is very close to the final experimental value (17%). The matching is almost exact if we exclude the 1% of tertiary amine used as catalyst that can be eluted with the sol. The slow variation of the sol fraction in a broad conversion predicted by the statistical model is also in qualitative agreement with experimental observations. 

**Figure 6:**
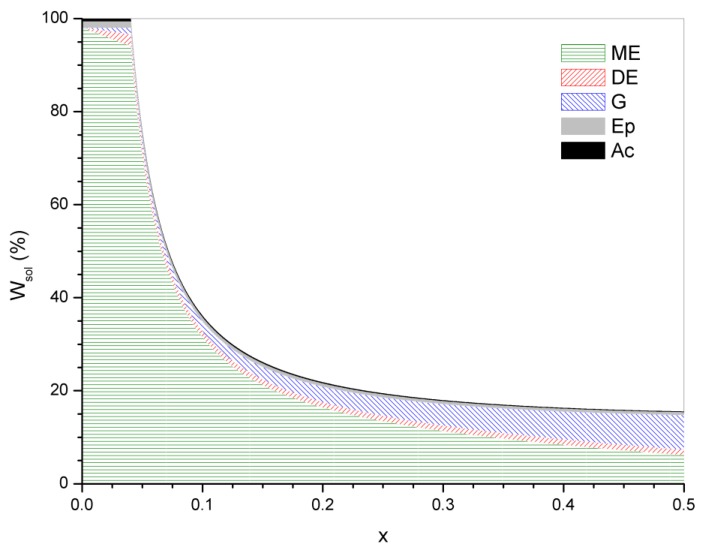
Contribution of different fragments to the sol fraction for x_E_ = 0.98.

The contribution of every fragment to the sol fraction is shown in Figure 6 for the case of x_E_ = 0.98. The contribution of the monoester fragment is the highest at low conversions while the contribution of the glycol becomes similar at conversions close to equilibrium.

## 4. Statistical Analysis of Transesterification in Stoichiometric A_3_ + B_2_ (Tricarboxylic Acid + Diepoxide) Formulations

Some formulations of epoxy vitrimers employ trifunctional carboxylic acids such as citric acid, tricarballylic acid and mixtures of dicarboxylic and tricarboxylic fatty acids (Pripol 1040). Although the fragment approach may be adapted to analyze any particular combination of polyfunctional monomers, we will restrict the discussion to the case of a formulation consisting of stoichiometric amounts of a tricarboxylic acid and a diepoxide.

In this case, we will assume that the epoxy-carboxylic acid reaction reached complete conversion (x_E_ = 1), before transesterification begins to take place. At this point, there is no sol fraction and all of the tricarboxylic acid fragments act as crosslinks of the network. We want to analyze the way in which transesterification modifies the statistical parameters of this ideal network.

**Figure 7:**
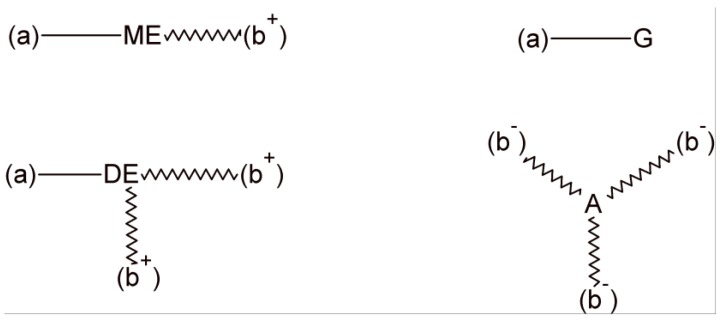
The four fragments of the polymer network during transesterification reactions. Bonds (a) represent half of a diepoxide molecule, bonds (b+) and (b−) represent (1/6) of a tricarboxylic acid molecule. The network is build-up by joining bonds (a) to bonds (a) and bonds (b+) to bonds (b−) with a probability determined by the concentration of the corresponding fragments.

Figure 7 shows the fragments generated during transesterification.

At any conversion (x) in the transesterification reaction, (ME) = (ME)_0_·(1 − x), where (ME)_0_ represents the concentration of the monoester fragments after completion of the epoxy-carboxylic acid reaction, (ME)_0_ = (Ep)_0_. The molar mass of the monoester fragment is equal to (M_E_/2) + (M_A_/6), where M_E_ is the molar mass of the diepoxide and M_A_ the molar mass of the tricarboxylic acid. The molar concentration of glycol is (G) = (ME)_0_·x/2. Its molar mass is equal to (M_E_/2) + 18. The molar concentration of diester is (ME)_0_·x/2. Its molar mass is equal to (M_E_/2) + (M_A_/3) − 18. The molar concentration of the acid skeleton is (A) = (ME)_0_/3 (corresponding to a 1:1 molar ratio of epoxy and carboxylic acid groups). Its molar mass is equal to M_A_/2.

After completion of the epoxy-carboxylic acid reaction and before the beginning of transesterification, only fragments (ME) and (A) are present in the system. The total mass is given by (ME)_0_(M_E_/2 + M_A_/6) + [(ME)_0_/3](M_A_/2) = (ME)_0_(M_E_/2 + M_A_/3). Mass fractions of every fragment during transesterification are then calculated as: W_ME_ = (1 − x)(M_E_/2 + M_A_/6)/(M_E_/2 + M_A_/3), W_G_ = (x/2)(M_E_/2 + 18)/(M_E_/2 + M_A_/3), W_DE_ = (x/2)(M_E_/2 + M_A_/3 − 18)/(M_E_/2 + M_A_/3), and W_A_ = (1/3)(M_A_/2)/(M_E_/2 + M_A_/3). It can be verified that the sum of all mass fractions equals 1.

Let us call F(a), F(b+) and F(b−) the probabilities of having finite continuations when looking out of bonds (a), (b+) and (b−), respectively.

(20) $$F\left(a\right)=\left(1-x\right)F\left(b+\right)+\frac{x}{2}+\frac{x}{2}F{\left(b+\right)}^{2}$$

(21) $$F\left(b+\right)=F{\left(b-\right)}^{2}$$

(22) $$F\left(b-\right)=\left(1-x\right)F\left(a\right)+2\frac{x}{2}F\left(a\right)\cdot F\left(b+\right)$$

By substituting Equation (22) into Equation (21) and using Equation (20), we get:

(23) $${\left[\left(1-x\right)F\left(b+\right)+\frac{x}{2}+\frac{x}{2}F{\left(b+\right)}^{2}\right]}^{2}{\left[1-x+x\cdot F\left(b+\right)\right]}^{2}-F\left(b+\right)=0$$

**Figure 8:**
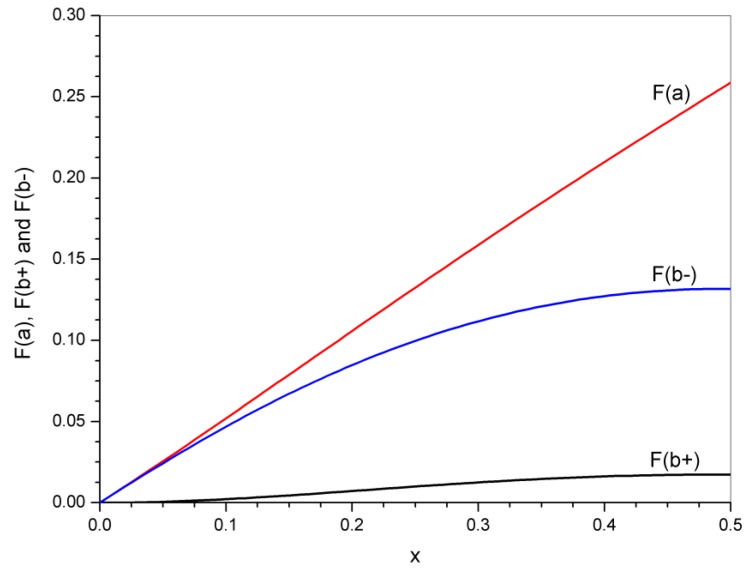
Evolution of probabilities F(a), F(b+) and F(b−) during transesterification of an A_3_ + B_2_ formulation.

A root for F(b+) comprised between 0 and 1 may be numerically found for any conversion x and values of F(a) and F(b−) may then be obtained from Equations (20) and (22), respectively. Figure 8 shows the variation of the three probabilities with x. For the equilibrium conversion, assumed as x_eq_ = 0.44, roots of Equations (20)–(22) are: F(b+) = 0.017, F(b−) = 0.1304 and F(a) = 0.2296.

The concentration of crosslinks at x = 0 was (A)/(ME)_0_ = (X_3_)/(ME)_0_ = 1/3. For any conversion during transesterification, the concentration of crosslinks is given by:

(24) $$\frac{\left(X_{3}\right)}{{\left(\mathrm{ME}\right)}_{0}}=\left(\frac{x}{2}\right)\left[1-F\left(a\right)\right]{\left[1-F\left(b+\right)\right]}^{2}+\frac{1}{3}{\left[1-F\left(b-\right)\right]}^{3}$$

**Figure 9:**
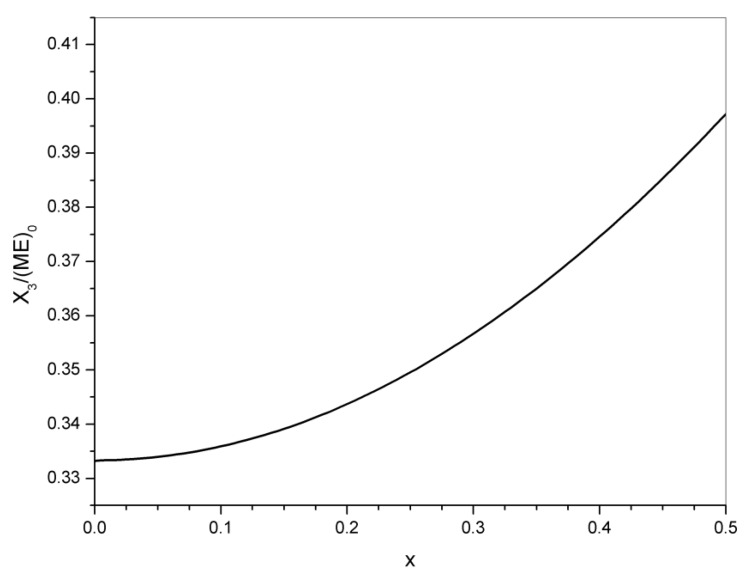
Evolution of the concentration of crosslinks during transesterification of an A_3_ + B_2_ formulation.

Figure 9 shows the increase in the concentration of crosslinks during transesterification reactions. 

For conversions close to equilibrium, assumed as x_eq_ = 0.44,

(25) $$\frac{\left(X_{3}\right)}{{\left(\mathrm{ME}\right)}_{0}}=0.164\;+\;0.219\;=\;0.383$$

Transesterification produced a 15% increase in the concentration of crosslinks, from the initial value of 0.333 to the final value of 0.383. A fraction of the initial crosslinks supplied by the tricarboxylic acid disappears but is compensated in excess by crosslinks arising from the diester fragments. Therefore, it is expected that the elastic modulus of the polymer network increases due to transesterification.

Pendant chains are also generated in the gel structure. Their concentration at equilibrium is given by (G)_eq_ [1 − F(a)], so that they reach a value close to 17% (ME)_0_. The presence of these chains will affect the viscoelastic properties of the network.

A sol fraction is also generated by transesterification. Just to provide an estimation of its value, we will consider a stoichiometric formulation of DGEBA (M_E_ = 340 g/mol) and tricarballylic acid (M_A_ = 176 g/mol). The sol fraction is given by:

(26) $$W_{\mathrm{sol}}=W_{\mathrm{ME}}\cdot F\left(a\right)\cdot F\left(b+\right)+W_{G}\cdot F\left(a\right)+W_{\mathrm{DE}}\cdot F\left(a\right)\cdot F{\left(b+\right)}^{2}+W_{A}\cdot F{\left(b-\right)}^{3}$$

**Figure 10:**
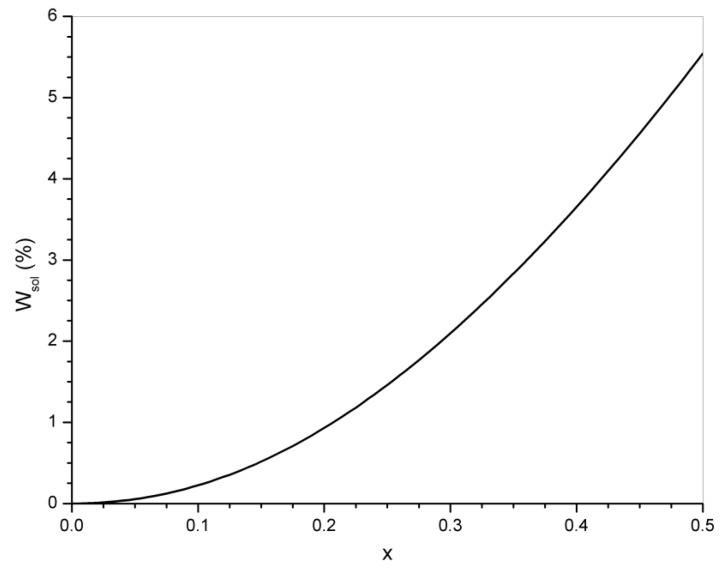
Evolution of the sol fraction during transesterification of an A_3_ (tricarballylic acid) + B_2_ (DGEBA) formulation.

Figure 10 shows the evolution of the sol fraction during transesterification. The mass fraction of sol at conversions close to equilibrium is 4.4% with the major contribution produced by glycol fragments (mainly DGEBA molecules with both ends converted to glycol groups).

## 5. Significance of the Statistical Analysis Regarding the Synthesis and Use of Epoxy Vitrimers

Most of the recent literature concerning epoxy vitrimers is focused on emphasizing the use of this relevant family of materials in advanced technological applications. The central role of bond exchange reactions on their properties is indicated but there is no insight into the effect of these reactions on the possible modification of statistical parameters of the polymer network. Our simple statistical analysis showed that, in the evolution towards an equilibrium state, transesterification reactions produce significant changes in the polymer structure. For the usual case of stoichiometric A_2_ + B_2_ formulations, transesterification is responsible for gelation (as shown by the group of Dušek more than three decades ago [38]), produced by the generation of crosslinks with an increasing concentration in the course of transesterification. As a counterpart, a relative large sol fraction remains when equilibrium is attained. For A_3_ + B_2_ formulations, chosen as an example of those producing gels during the previous epoxy-carboxylic acid reaction, transesterification reactions increase the crosslink density and simultaneously generate pendant chains and a sol fraction.

Transesterification reactions are very much slower than epoxy-carboxylic acid reactions (they require long times at high temperatures). Therefore, unless the synthesis of the epoxy vitrimer is performed including a prolonged final step at a high temperature, transesterification could be out of equilibrium. In this case, during the use of the vitrimer at T > T_v_, the network structure will continuously change towards the equilibrium. As the equilibrium constant might change with temperature, the structure could still be modified by employing different temperatures in this range. The evolution of the elastic modulus of the epoxy vitrimer in the rubbery state can be used as an indicator of the attainment of equilibrium in transesterification reactions. Just to mention an example from the literature, an epoxy vitrimer based on DGEBA and Pripol 1040, synthesized by heating at 130 °C for 6 h, had an elastic modulus of 3.5 MPa at 23 °C [15]. Heating for 1 h at 180 °C increased the elastic modulus to 3.7 MPa[15], a fact that might be assigned to the advance of transesterification reactions. Possibly, a further increase could have been observed if a prolonged heating at 180 °C had been performed.

## 6. Conclusions

A statistical analysis based on the fragment approach was performed to analyze the evolution of the network structure of epoxy vitrimers during transesterification reactions. An analytical solution enabling the calculation of statistical parameters in the postgel stage was obtained for a formulation based on a diepoxide and a dicarboxylic acid. A numerical solution was presented for the reaction of a diepoxide with a tricarboxylic acid, as an example of the way to apply the model to polyfunctional monomers. As transesterification acts as a disproportionation reaction that converts two linear fragments (monoesters) into a terminal fragment (glycol) and a branching fragment (diester), its effect is to increase the concentration of crosslinks and pendant chains while leaving a sol fraction. Changes in the network structure of the epoxy vitrimer can take place after their synthesis, during their use at high temperatures, a fact that has to be considered in their technological applications.
